# Development of a new quantitative RT-PCR to detect lymphocytic choriomeningitis virus

**DOI:** 10.3389/fvets.2025.1651039

**Published:** 2025-12-24

**Authors:** Laura Herrero, Nuria Labiod, Francisca Molero, Arantxa Potente, María Paz Sánchez-Seco, Ana Vázquez

**Affiliations:** 1 Centro Nacional de Microbiología, Instituto de Salud Carlos III, Madrid, Spain; 2 Centro de Investigación Biomédica en Red de Enfermedades Infecciosas (CIBERINFEC), Instituto de Salud Carlos III, Madrid, Spain; 3 Ciber de Epidemiología y Salud Pública (CIBERESP), Instituto de Salud Carlos III, Madrid, Spain

**Keywords:** LCMV, arenaviruses, rodent-borne diseases, RT-qPCR, viral diagnosis

## Abstract

Lymphocytic choriomeningitis virus (LCMV) is a neglected rodent-borne virus, with a worldwide distribution. The common mouse *Mus musculus* acts as reservoir and vector in the biological cycle of the virus. Surveillance of LCMV infection in mice is of importance as they are a widely used animal model in research and, through contact with them or their fluids, humans can be infected. Although most human cases are asymptomatic, LCMV infection can cause mild to severe, even fatal, and new diagnostic tools need to be developed to improve its detection. In the present work we report the development of a new method for the detection of LCMV RNA by quantitative reverse transcription polymerase chain reaction (RT-qPCR), able to detect all LCMV strains described to date. RT-qPCR targeting the S segment was developed and evaluated. Specificity and sensitivity were determined, and its limit of detection (LOD) was defined. The method designed is able to detect all 5 LCMV lineages described to date, with a LOD of 5.6 genome copies/μL. Its design with a built-in internal amplification control allows the detection of false negative results. Other arenaviruses were found not to cross-react with the method designed. In conclusion, a new diagnostic RT-qPCR for the detection of LCMV have successfully designed and validated. Improved detection techniques allow to reduce the turnaround time in the diagnosis of infections and to improve epidemiological surveillance in humans and animals.

## 1 Introduction

Lymphocytic choriomeningitis virus (LCMV) is a zoonotic agent, which belongs to the *Arenaviridae* family. This family includes 5 genera, but only the genus *Mammarenavirus* has been described as competent to infect mammals, including humans (1). Rodents are recognized as the main natural reservoirs of the genus, although some mamarenaviruses have been isolated from fruit-eating bats (2, 3). To date, two major groups have been described within *Mammarenavirus* genus based on the geographical distribution of the viruses and their rodent reservoirs, the Old World Group (OWG) and the New World Group (NWG) (4). LCMV has the widest known distribution of all *Mammarenaviruses*, being predominant in North America and Europe. This wide distribution is explained by the geographical distribution of its main host and reservoir, the mouse *Mus musculus*, which is present in all continents except Antarctica and the North Pole (5, 6). Other reservoirs of the virus, and therefore potential transmitters, are the field mouse (*Apodemus sylvaticus*), the fawn mouse (*Apodemus flavicolis*), hamsters and guinea pigs (*Cavia porcellus*) (7–9).

LCMV is an enveloped, segmented single-stranded, ambisense RNA genome, whose segments are called L (large) and S (small) (10, 11). Considering the genetic diversity of LCMV S-segment, five lineages (Lineage I, II, III, IV and V) have been described (7, 8, 12, 13). Lineage I is the most widespread and includes most of the described strains, such as the classical laboratory strains Armstrong and WE.

In their natural host, rodents, LCMV transmission usually occurs by inhalation of aerosols generated from contaminated droppings (saliva, semen, milk, urine and faeces) and typical social behaviors of rodents, such as grooming, favor transmission. In addition, the virus can be transmitted vertically to subsequent generations by intrauterine infection, resulting in a generation of persistently infected mice that will excrete the virus throughout their lives (11, 14, 15). Because they usually exhibit no overt clinical signs, the infection can spread silently through high-density colonies typical of laboratory animal facilities. As a result, personnel who handle or work near these animals—including animal care technicians, veterinarians, and laboratory staff—are at increased risk of occupational exposure to this zoonotic pathogen (16, 17). Humans can be infected by inhalation of infected excreta that may be aerosolised during sweeping or cleaning mice (18) or by direct contact with contaminated material. Animal bites, ingestion of contaminated food and solid organ transplants are another potential route of LCMV infection (11, 19–22). Most infections are asymptomatic, or only a mild, self-limiting febrile illness develops. However, in severe cases, after a temporary improvement of these flu-like symptoms, a second phase of central nervous system involvement usually follows. The symptoms of this second phase are usually those of classical aseptic meningitis or meningoencephalitis (23, 24). Other LCMV-related complications include hydrocephalus, pancreatitis, orchitis, arthritis, parotitis, and pericarditis (11, 25, 26). LCMV can also be transmitted vertically from mother to foetus via the transplacental route (27, 28). In congenital LCMV infection the mortality rate ranges from 14 to 35% depending on the time of infection (23, 29). When infants survive, congenital malformations such as chorioretinitis, hydrocephaly, psychomotor retardation, microcephaly or ventriculomegaly are common (23, 30–32).

At present, no specific vaccine or treatment against LCMV is available either in humans or in rodents. Therefore, the cornerstone of disease control strategies should be based on regular health surveillance and good diagnosis of infection. Laboratory diagnosis of LCMV infections can be approached in two ways: using direct or indirect methods. Acute LCMV infections can be diagnosed by viral isolation or by conventional PCR assays from samples such as serum, blood, urine, cerebrospinal fluid, organs and throat swabs (33, 34). Serological methods, ELISA and immunofluorescence assay (IFA) are used to detect IgM and IgG antibodies in serum samples, although very few commercial tests are currently available (14, 35).

Based on these concerns, the present study describes the development and validation of a novel RT-qPCR assay for the detection of LCMV, designed to enhance diagnostic capacity and animal surveillance and occupational health protection settings.

## 2 Materials and methods

### 2.1 Primer and probes design

LCMV specific primers and probe were designed in the nucleoprotein (NP) region of the S segment. For this purpose, an extensive alignment of 34 LCMV sequences, representative of all LCMV lineages described to date, was performed. All sequences were available in the NCBI GenBank database (National Center for Biotechnology Information) (Table 1). Primers and probe were designed using Primer Express® v2.0 software (Applied Biosystems, Foster City, CA, USA). Primers were also analysed with the Oligo Primer Analysis v6.0 program (Molecular Biology Insights).

**Table 1:** Sequences used in the design of RT-qPCR oligonucleotides for the detection of lymphocytic choriomeningitis virus.

Strain	GenBank accession number	Host	Country	Isolate date	Lineage
Armstrong 53b	AY847350	*H. sapiens*	USA	1933	I
OQ28	AB627952	*M. musculus*	Japan	1990	I
WE Nagasaki	AB627951	*H. sapiens*	Japan	2011	I
LCM	DQ118959	*M. musculus*	USA	1977	I
LCM	DQ286931	*M. musculus*	France	2004	I
CH-5692	DQ868483	Unpublished	France	2009	I
Pasteur	DQ868485	Unpublished	France	1977	I
Traub	DQ868487	*M. musculus*	Germany	1960	I
Aggressive	EU480450	NR	USA	1970	I
Docil	EU480452	NR	USA	1970	I
810366	FJ607028	*H. sapiens*	USA	2003	I
HP65-2009/1	JF912085	*M. musculus*	France	2009	I
201102714	JN687949	*H. sapiens*	USA	2009	I
200501927	FJ607030	*C. cricetus*	USA	2005	I
811316	FJ607031	*H. sapiens*	USA	2008	I
810885	FJ607032	*M. musculus*	USA	2005	I
NY06-WHI5107	FJ607033	*H. sapiens*	USA	1949	I
NY09-WE-UBCA337	FJ607034	*M. musculus*	USA	1935	I
NY38-Douglas4707	FJ607035	*H. sapiens*	USA	1947	I
WE UBC57135	FJ607036	*M. musculus*	USA	1935	I
200504261	FJ607037	*C. cricetus*	USA	2005	I
810362	FJ607038	*H. sapiens*	USA	2003	I
IN-2012 (M435)	KF732824	*M. musculus*	USA	2012	I
Makokou	KM523323	*M. musculus*	Gabon	2012	I
WE New York	M22138	*H. sapiens*	USA	1935	I
Bulgaria	GQ862982	*NR*	Bulgary	1956	II
SK1042	MZ558311	*M. musculus*	Czech Republic	2009	II
KS22-098_L5719	OP958782	*M. musculus*	Germany	2022	II
211011664_L5409	OP958778	*L. rosalia*	Germany	2022	II
810935	FJ607029	*H. sapiens*	USA	1984	III
CABN	FJ895882	*A. sylvaticus*	Spain	2004	IV
GR01	FJ895883	*A. sylvaticus*	Spain	2004	IV
SN05	FJ895884	*A. sylvaticus*	Spain	2004	IV
KS20-3119	OR135709	*A. sylvaticus*	Germany	2020	V

NR, not reported; *H. sapiens*, *Homo sapiens*; *C. cricetus*, *Cricetus cricetus*; *L. rosalia*, *Leontopithecus rosalia*.

Once designed, primers and probe were introduced into a second alignment, containing 24 different sequences of several *Mammarenaviruses* (Table 2), in order to perform an *in silico* specificity study, and to check that the sequences designed for the primers and probe in the chosen region did not have sufficient homology to be able to amplify other viruses of the same genus (36). Two different TaqMan® probes were designed, one specific LCMV probe (LCMV_probe) and an internal control specific probe (IC_Probe), labelled at the 5′-end with FAM and NED, respectively. MGB (Minor Groove Binder) and NFQ (Non-Fluorescent Quencher) were used at the 3′-ends (Table 3).

**Table 2:** Sequences of viruses belonging to the *Mammarenavirus* genus used to study *in silico* the specificity of the developed LCMV RT-qPCR.

Virus	Group	GenBank accession number	Isolation host	Country of isolation	Isolation date
Chapare	NW	NC_010562	*H. sapiens*	Bolivia	2003
Chapare	NW	EU260463	*H. sapiens*	Bolivia	2003
Flexal	NW	AF485257	NR	Brazil	NR
Flexal	NW	U43687	NR	NR	NR
Guanarito	NW	AY497548	*H. sapiens*	Venezuela	NR
Guanarito	NW	AY572554	*H. sapiens*	Venezuela	NR
Junín	NW	KR260734.1	*H. sapiens*	Australia	1977
Junín	NW	KU978800.1	*H. sapiens*	Argentina	1991
Machupo	NW	KU978794.1	*H. sapiens*	Bolivia	1994
Machupo	NW	KU978792.1	*H. sapiens*	Bolivia	2011
Pichinde	NW	AF081555	*Cavia porcellus*	NR	NR
Pichinde	NW	JN378747	*Cavia porcellus*	USA	2009
Sabiá	NW	JN801474	Vero E6	USA	1996
Sabiá	NW	U41071	Vero E6	Brazil	1996
Tacaribe	NW	KF923400	*A. americanum*	USA	2012
Tacaribe	NW	NC_004293	Recombinant	USA	2018
Whitewater Arroyo	NW	AF228063	Vero E6	USA	2001
Whitewater Arroyo	NW	AF485264	Vero E6	USA	2002
Lassa	OW	GU481064	*H. sapiens*	Nigeria	2008
Lassa	OW	PP826286.1	*H. sapiens*	Sierra Leone	2012
Lujo	OW	FJ952384	*H. sapiens*	South Africa	2008
Lujo	OW	JX017360	Recombinant	USA	2012
Mopeia	OW	M33879	NR	Mozambique	1991
Wenzhou	OW	KM051422	*Sorex araneus*	China	2014

NR, not reported; NW, new world arenavirus; OW, old world arenavirus; *H. sapiens*, *Homo sapiens*.

**Table 3:** Primers and probes designed for LCMV and IC detection.

Name	Sequence (5′–3′)	Nucleotide positions
LCMV TR-F	AGTCCATRAGNGCRCARTG	1737–1755
LCMV TR-Re	GTGTGGGACAANTAYGGNT	2,687–2,705
LCMV_probe	FAM-CCNGTRTGCATYTTRCANA-MGB-NFQ	1793–1811
IC_Probe	NED-CCAGCACACATGTGTCTACT-MGB-NFQ	

Positions based on the complete sequence of the S-segment from LCMV Armstrong 53b strain (GenBank, acc. no. AY847350).

### 2.2 Design of internal control plasmid and DNA standard

To detect possible inhibitions in the PCR reaction, an internal control (IC) was designed. A method like that described by other authors was used (37–39), consisting of the synthesis of a plasmid DNA fragment containing a known sequence of the BK virus, flanked by the sequence of the primers designed in this work for LCMV amplification in the RT-qPCR (Table 4). To generate a plasmid DNA-based standard curve, primers designed for RT-qPCR were used to amplify part of the S fragment of the LCMV Amstrong strain (GenBank: AY847350). PCR products were purified and cloned using TOPO TA Cloning® kit (Invitrogen, MA, USA) according to the manufacturer’s instructions. Plasmids obtained were sequenced to confirm the absence of mutations, quantified by measuring the optical density at 260 nm using a NanoDrop spectrophotometer (Thermo Fisher Scientific, USA), and the copy number was calculated using the formula of Reed-MuenchQuantified DNA was linearized using the Not I restriction enzyme (New England Biolabs, MA, USA) and, for LCMV standard DNA, a 10-fold dilution curve was constructed from 10^6^ to 10^1^ DNA copies/μL using water as diluent.

**Table 4:** Primers used for internal control construction.

Name	Sequence (5′–3′)
IC-F	AGTCCATRAGNGCRCARTG**CCAGCACACATGTGTCTACT**
IC-Re	GTGTGGGACAANTAYGGNT**AGTAGACACATGTGTGCTGG**

Overlapping nucleotides are marked in bold and correspond to the sequence of the IC probe.

### 2.3 RNA extraction

The nucleic acids used in this work were extracted using the commercial kit QIAamp® Viral RNA (Qiagen, Hilden, Germany) following the manufacturer’s recommendations. For RT-qPCR optimisation, a RNA extract (Armstrong strain 53b; GenBank: AY847350) from a LCMV isolated in Vero E6 cells was used as template.

### 2.4 Real time RT-PCR assay

The RT-qPCR reaction was carried out in a 7,500 Fast Real-Time PCR system (Applied Biosystems), using the commercial kit QuantiTect® Multiplex RT-PCR Kit (Qiagen, Hilden, Germany). For each assay, 5 μL of RNA sample was mixed with 20 μL of a reaction mix containing: 3.45 μL of sterile water, 12.5 μL of Quantitec multiplex RT-PCR master, 0.25 μL of each primer (100 μM), 0.75 μL of LCMV probe (10 μM), 1.5 μL of IC probe (10 μM), 1 μL of IC (10^2^ copies/μL) and 0.3 μL of RT mix. Amplification parameters were established as follows: an initial retrotranscription step at 50 °C for 30 min, a DNA polymerase activation cycle at 95 °C for 15 min, followed by 45 amplification cycles at 94 °C for 45 s and 60 °C for 1 min. Fluorescence measurement for FAM and NED was performed simultaneously at the end of each cycle. Cycle threshold values (Ct) were measured as the point at which the fluorescence signal of the sample fluorescence crossed a predetermined threshold value.

### 2.5 Evaluation of sensitivity and specificity of the qRT-PCR

To assess the sensitivity of RT-qPCR, standard plasmids were used as template. Each reaction was replicated four times in the same assay, and the tests were performed on three different days. The LOD was calculated by adaptation of the Reed–Muench formula (40). Specificity was assessed by testing a panel of 28 arenaviruses genomes and other encephalitis-producing viruses belonging to other viral families (Supplementary Table S1).

### 2.6 Evaluation of repeatability (intra-assay precision) and reproducibility (inter-assay precision)

The repeatability and reproducibility of the RT-qPCR assay were determined using six different concentrations (10^6^, 10^5^,10^4^, 10^3^, 10^2^ and 10 copies/μL) of the standard plasmids. Quintuplicate analysis of each dilution were performed on the same day to determine intra-assay variability. Additionally, three different experiments were performed in different days to assess inter-assay variability. The coefficient of variation of the Ct values was determined based on the intra-assay and inter-assay results.

### 2.7 Evaluation of the possible application of the real-time PCR method using preparation of synthetic clinical samples

Due to the absence of LCMV PCR-positive human clinical samples in our laboratory, a panel of 15 simulated human clinical samples was constructed to evaluate this RT-qPCR. Six known negative samples were also included in the panel. The simulated clinical samples, urine, blood or serum, were generated by inoculating 20 μL of a titled LCMV viral culture (Armstrong strain 53b GenBank: AY847350) into 180 μL of the clinical sample to finally obtain samples containing different concentrations of viral genome (10^4^–1 copies of viral genome per microlitre). These clinical samples had previously been tested for LCMV RNA to ensure negativity using a conventional nested RT-PCR adapted from Ledesma et al. (8) by using LCMV sequences instead of degenerations (Supplementary Table S2). Viral inactivation and RNA extraction were performed using standard procedures as described above.

## 3 Results

### 3.1 Design of primers, probes and standard curve for the RT-qPCR assay

Primers and probes targeting a highly conserved fragment of the LCMV S-segment amplifying a 96 bp sequence were designed.

**Figure 1:**
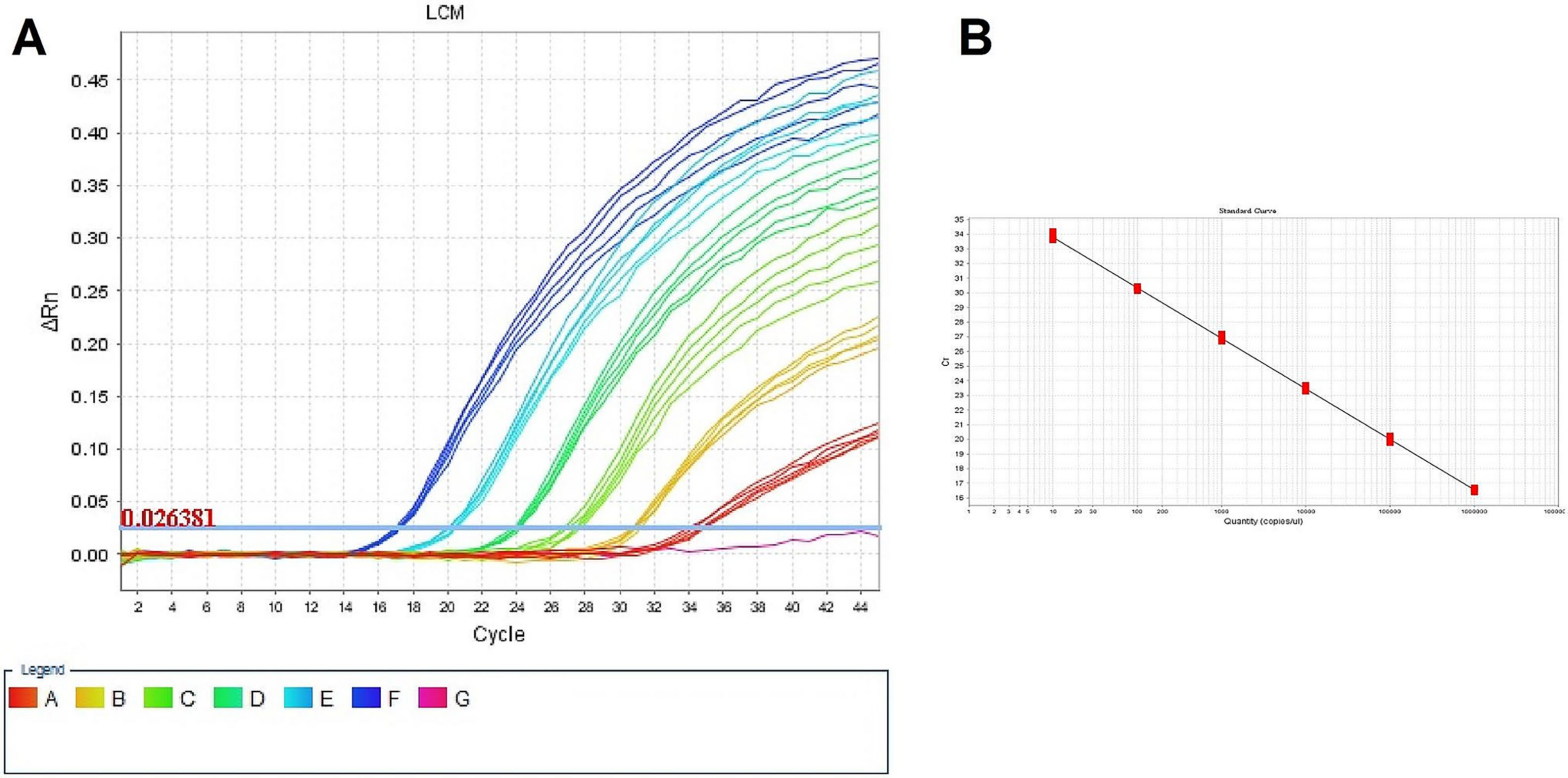
Amplification for the LCMV DNA standard. The copy number of the standard plasmid ranged 10^6^ (F), 10^5^ (E), 10^4^ (D), 10^3^ (C), 10^2^ (B), 10^1^ (A) and 10^1^ (G) copies/μL. **(A)** Amplification curve for LCMV target representing fluorescence increase vs. cycle. **(B)** Standard curve representing Ct vs. DNA quantity.

The standard curves for RT-qPCR were constructed using a 10-fold dilution series ranging from 10^6^ to 10 copies/μL of the corresponding DNA standard in each reaction. The standard curve for RT-qPCR showed that the slope is −3.448, the amplification efficiency (Eff%) is 95.006 and that the correlation coefficient *R*^2^ is 0.999 (Figure 1). Both *R*^2^ and efficiency values indicate a strong linear relationship between the template and the Ct value, validating the reliability of the newly developed RT-qPCR assay.

### 3.2 Sensitivity and specificity of real-time RT-PCR

The LOD was assessed using serial dilutions of the quantified LCMV DNA standard the sensitivity limit of this RT-qPCR using the Reed–Muench formula is 5.6 copies/μL. To assess specificity, different viral genomes were used as templates of the LCMV RT-qPCR. No amplification curves were detected for any other viruses, demonstrating that the developed RT-qPCR method does not cross-react neither with other encephalitis-producing viruses nor with other arenaviruses.

### 3.3 Repeatability and reproducibility of the real-time PCR assay

The repeatability and reproducibility of the RT-qPCR assay were evaluated by testing different concentrations of the LCMV standard plasmids. The relationship between the standard deviation (SD) and the average (X̅) obtained for the Ct values in five replicates of each DNA standard concentration, was calculated using the coefficient of variation (CV). Intra-assay CV values ranged from 0.39 to 1.1% and between assays from 0.90 to 4.59% for the limit dilution (Table 5). These results indicated a satisfactory repeatability and reproducibility for the developed RT-qPCR assay.

**Table 5:** Repeatability and reproducibility RT-qPCR assay for detection of LCMV.

Template (copies/μl)	Intra-coefficient of variation		Inter-coefficient of variation	
	X̅ ± SD	CV (%)	X̅ ± SD	CV (%)
1,000,000	16.58 ± 0.14	0.86	16.64 ± 0.17	1.00
100,000	19.98 ± 0.22	1.10	20.01 ± 0.19	0.94
10,000	23.48 ± 0.19	0.81	23.59 ± 0.21	0.90
1,000	26.90 ± 0.21	0.79	26.99 ± 0.28	1.03
100	30.28 ± 0.12	0.39	30.14 ± 0.19	0.64
10	33.85 ± 0.26	0.77	34.19 ± 1.57	4.59

The average (X̅), the standard deviation (SD) and the coefficient of variation (CV) of the Ct values obtained for each template quantity are shown.

### 3.4 Detection in synthetic clinical samples

**Figure 2:**
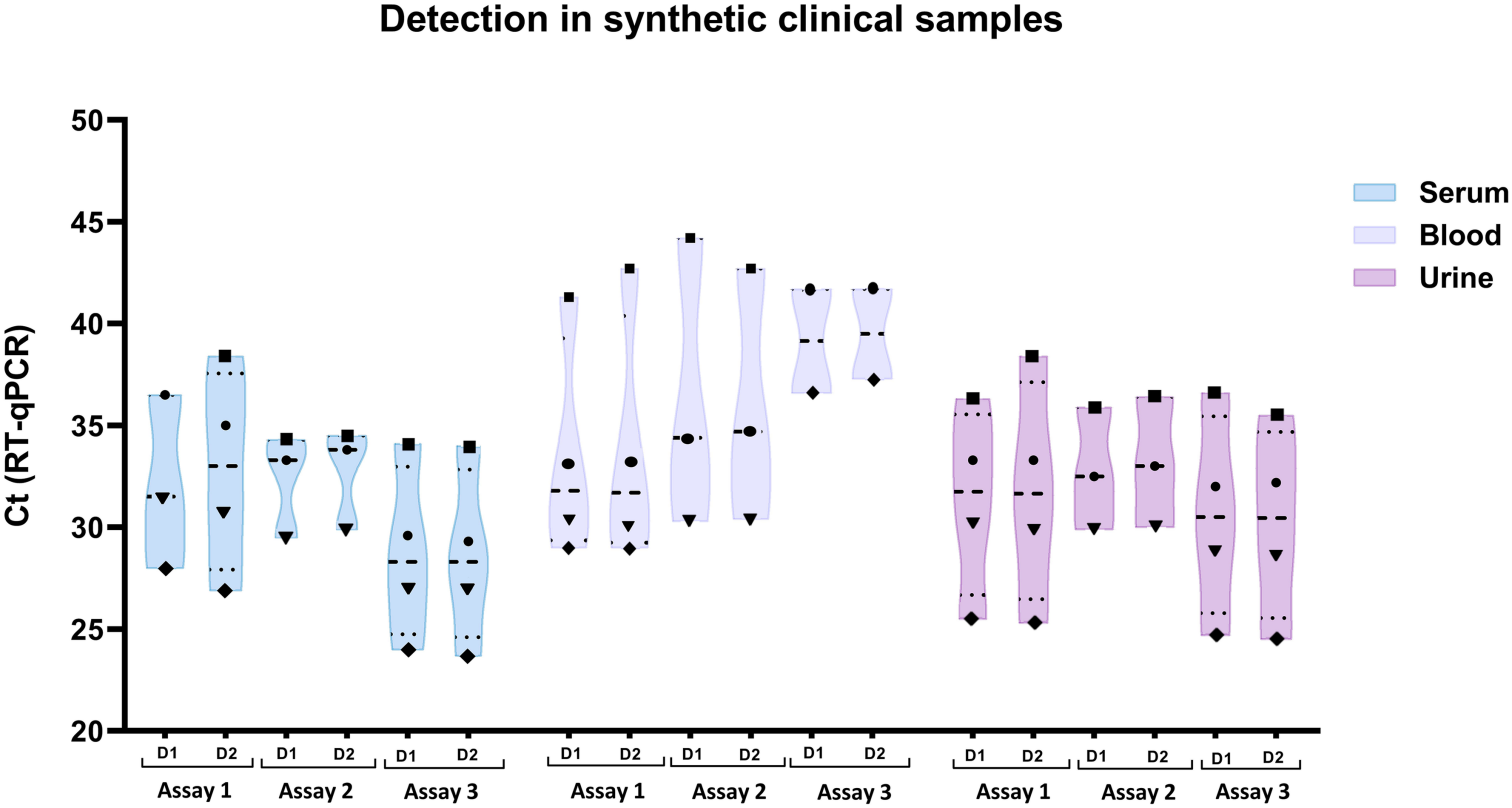
Detection of LCMV RNA in synthetic clinical samples by RT-qPCR. Cycle threshold (Ct) values obtained for serum, blood, and urine samples spiked with LCMV at different concentrations are shown. Each sample type was tested across three independent experimental assays (Day 1–3), with duplicate reactions (D1, D2) performed on each day. Symbols correspond to the viral genome concentration used for spiking: 10^1^ copies/μL (■); 10^2^ copies/μL (●); 10^3^ copies/μL (▼) and 10^4^ copies/μL (♦). The assay consistently detected viral RNA down to 10 genome copies/μL in all sample types, with Ct values ranging from ~30 to 44, whereas samples spiked with 1 copy/μL were not detected (see Table 6).

The results of the simulated clinical samples showed that this RT-qPCR (Figure 2) was able to detect LCMV RNA with a concentration of 10 copies of viral genome/μL, in 15 of the 16 samples tested, with Ct values between 34 and 44, depending on the sample type. Samples contaminated with 1 copy of viral genome/μL were negative in all assays (Table 6).

**Table 6:** LCMV detection in synthetic clinical samples.

			Non infected	Viral genome (copies/μL)				
				10^0^	10^1^	10^2^	10^3^	10^4^
Serum	Day 1	RT-qPCR	Nd	Nd	Nd	36.5	31.5	28
		RT-qPCR	Nd	Nd	38.4	35	31	26.9
		Nested RT-PCR		Nd	+	−	+	+
	Day 2	RT-qPCR		Nd	34.3	33.3	29.5	
		RT-qPCR		Nd	34.5	33.8	29.9	
		Nested RT-PCR		Nd	+	+	+	+
	Day 3	RT-qPCR		Nd	34.1	29.6	27	24
		RT-qPCR		Nd	34	29.3	27.3	23.7
		Nested RT-PCR		+	+	+	+	+
Blood	Day 1	RT-qPCR	Nd	Nd	41.3	33.2	30.4	29
		RT-qPCR	Nd	Nd	42.7	33.4	30	29
		Nested RT-PCR		Nd	Nd	+	+	+
	Day 2	RT-qPCR		Nd	44.2	34.4	30.3	
		RT-qPCR		Nd	42.7	34.7	30.4	
		Nested RT-PCR		Nd	+	+	+	
	Day 3	RT-qPCR		Nd	Nd	41.7	Nd	36.6
		RT-qPCR		Nd	Nd	41.7	Nd	37.3
		Nested RT-PCR		Nd	Nd	Nd	Nd	Nd
Urine	Day 1	RT-qPCR	Nd	Nd	36.3	33.3	30.2	25.5
		RT-qPCR	Nd	Nd	38.4	33.3	30	25.3
		Nested RT-PCR		+	+	+	+	+
	Day 2	RT-qPCR		Nd	35.9	32.5	29.9	
		RT-qPCR		Nd	36.4	33	30	
		Nested RT-PCR		+	+	+	+	
	Day 3	RT-qPCR		Nd	36.6	32	29	24.7
		RT-qPCR		Nd	35.5	32.2	28.7	24.5
		Nested RT-PCR		+	+	+	+	+

Samples were tested in RT-qPCR by duplicate and the presence or absence of viral RNA was confirmed by and the end-point nested RT-PCR described in the article. Nd, not detected.

## 4 Discussion

Lymphocytic choriomeningitis virus (LCMV) is a pathogen whose life cycle is associated with the distribution and activity of rodent populations. In wild mouse populations, studies show prevalences ranging from 0 to 25%, depending on the region and sampling conditions (41–44). In other mammals, LCMV infections are not uncommon (13, 45) and can even infect humans (24, 46, 47). Human infections are underestimated and little studied, but in Europe there are studies showing human seroprevalences of 6.8% in Croatia (48), 7% in Italy (49) and 1.7–2% in Spain (50, 51). All these data indicate that LCMV is currently circulating in our environment. In recent decades, climate change has caused global temperatures to rise, making rodents able to reproduce faster and more efficiently (52, 53). It has also led to changes in vegetation or precipitation that have caused rodent populations to seek refuge indoors. This has changed the behavior of rodent populations, making them more likely to come into contact with humans and increasing the transmission of zoonotic diseases (54, 55).

On the other hand, a significant relationship has been found to exist between rodent population density and the prevalence of LCMV infection (48). And that within infested households, LCMV infection rates in mice range from 50% to almost 100% (34, 48), increasing the risk of LCMV infection in humans living there. This increased transmission of LCMV when there is a high density of rodents may be of particular importance in animal breeding and experimental animal facilities, where, in addition to the health of the animals, the health of the workers may be affected. In 1972–1973 at the University of Rochester Medical Centre, an outbreak of LCMV was reported among 48 infected workers, caused by Syrian hamsters used in tumour research (56). In 2012 there was an LCMV outbreak in several EEUU rodent facilities, and a third of the employees were infected, developing, some of them, aseptic meningitis (57). Animal health surveillance in these facilities is essential to ensure both the health of animals and workers, as the absence of microorganisms that could interfere with research. Federation of European Laboratory Animal Science Associations, US Department of Health and Human Services recommend the implementation of surveillance programmes to detect and control potential pathogens, including LCMV (58–60). Although traditional pathogen detection methods such as necropsies remain powerful tools for the diagnosis of infectious diseases in animals, molecular methods significantly improve the sensitivity of the tests. For that purpose, we describe a new RT-qPCR assay for LCMV detection that would be suitable for rapid and specific diagnostic objectives. Currently, several conventional nested RT-PCRs have been published for the molecular diagnosis of LCMV infection, which involve multiple time-consuming steps and are associated with an increased risk of contamination. Few quantitative PCRs have been described to date. Of the published designs, some require a previous cDNA synthesis step (61, 62), and others are SYBR Green quantitative PCRs (34). Extensive efforts during the SARS-CoV-2 pandemic have focused on the validation and standardization of RT-PCR assays. These studies highlighted the need for assay optimization, sensitivity and specificity assessment, inter-laboratory comparability, and harmonized validation protocols. Lessons learned from SARS-CoV-2 diagnostics provide valuable benchmarks to strengthen molecular diagnostic strategies for other viral pathogens (63, 64). The RT-qPCR developed in this study is a one-step PCR theoretically capable of detecting all five LCMV lineages described to date. Its design is based on hydrolysis probes (Taqman probes), which increases specificity and reproducibility in the detection of PCR amplification products, compared to other qPCR chemistries. In addition, an internal control was also designed to be co-amplified in each reaction mixture to aid detection of false negative results due to lack of amplification for example by polymerase inhibition. Other features of this new RT-qPCR are a low detection limit, a high ratio of genomic load to Ct and consistently repeatability and reproducibility. Using synthetic clinical samples, the efficacy of this new PCR could be evaluated, since it was able to detect the presence of LCMV in all three types of synthetical clinical samples tested: serum, blood and urine. Using the same synthetic clinical samples, the accuracy and stability of the new PCR was also validated by comparison with a nested PCR commonly used in the laboratory for LCMV detection. The results showed that the detection limit of the new RT-qPCR in clinical samples was between 10 and 1 copies/μL, which was in line with the detection limit obtained when using standard DNA (5.6 copies/μL). Moreover, this technique was validated using RNA obtained from mouse samples. The evaluation of the specificity of RT-qPCR was performed using genomes of viruses belonging to *Arenaviridae* or different families, all of them producing the same or similar symptoms. The absence of amplification signal in these samples demonstrated the high accuracy of the method developed for LCMV detection, as it only detects the genome of the virus for which it has been designed. The diagnosis of infectious diseases in clinical samples requires the use of specific, highly sensitive and reproducible techniques. The RT-qPCR designed in this work provides these characteristics, guarantees the detection of all LCMV lineages circulating to date without cross-reaction with other arenaviruses or other encephalitis-producing viruses and, in addition, avoids false-negative results.

## Data Availability

The datasets presented in this study can be found in online repositories. The names of the repository/repositories and accession number(s) can be found in the article/Supplementary material.

## References

[1] Radoshitzky SR Buchmeier MJ Charrel RN Gonzalez J-PJ Günther S Hepojoki J . ICTV virus taxonomy profile: arenaviridae 2023. J Gen Virol. (2023) 104:001891. doi: 10.1099/jgv.0.001891, PMID: 37698490 PMC10720992

[2] Malmlov A Seetahal J Carrington C Ramkisson V Foster J Miazgowicz KL . Serological evidence of arenavirus circulation among fruit bats in Trinidad. PLoS One. (2017) 12:e0185308. doi: 10.1371/journal.pone.0185308, PMID: 28953976 PMC5617188

[3] Bentim Góes LG Fischer C Almeida Campos AC de Carvalho C Moreira-Soto A Ambar G . Highly diverse arenaviruses in neotropical bats, Brazil. Emerg Infect Dis. (2022) 28:2528–33. doi: 10.3201/eid2812.220980, PMID: 36417964 PMC9707603

[4] Radoshitzky SR Bào Y Buchmeier MJ Charrel RN Clawson AN Clegg CS . Past, present, and future of arenavirus taxonomy. Arch Virol. (2015) 160:1851–74. doi: 10.1007/s00705-015-2418-y, PMID: 25935216

[5] Childs JE Peters CJ. Ecology and epidemiology of arenaviruses and their hosts. In: Salvato MS, editor. The arenaviridae. Boston, MA: Springer US (1993). 331–84. doi: 10.1007/978-1-4615-3028-2_19

[6] Lendino A Castellanos AA Pigott DM Han BA. A review of emerging health threats from zoonotic new world *Mammarenaviruses*. BMC Microbiol. (2024) 24:115. doi: 10.1186/s12866-024-03257-w, PMID: 38575867 PMC10993514

[7] Mehl C Adeyemi OA Wylezich C Höper D Beer M Triebenbacher C . Lymphocytic choriomeningitis virus lineage V in wood mice, Germany. Emerg Infect Dis. (2024) 30:399–401. doi: 10.3201/eid3002.230868, PMID: 38270110 PMC10826776

[8] Ledesma J Fedele CG Carro F Lledó L Sánchez-Seco MP Tenorio A . Independent lineage of lymphocytic choriomeningitis virus in wood mice (*Apodemus sylvaticus*), Spain. Emerg Infect Dis. (2009) 15:1677–80. doi: 10.3201/eid1510.090563, PMID: 19861074 PMC2866409

[9] Skinner HH Knight EH Buckley LS. The hamster as a secondary reservoir host of lymphocytic choriomeningitis virus. J Hyg. (1976) 76:299–306. doi: 10.1017/S0022172400055194, PMID: 1063218 PMC2129631

[10] Salvato MS Shimomaye EM. The completed sequence of lymphocytic choriomeningitis virus reveals a unique RNA structure and a gene for a zinc finger protein. Virology. (1989) 173:1–10. doi: 10.1016/0042-6822(89)90216-X, PMID: 2510401

[11] Lapošová K Pastoreková S Tomášková J. Lymphocytic choriomeningitis virus: invisible but not innocent. Acta Virol. (2013) 57:160–70. doi: 10.4149/av_2013_02_160, PMID: 23600874

[12] Albariño CG Palacios G Khristova ML Erickson BR Carroll SA Comer JA . High diversity and ancient common ancestry of lymphocytic choriomeningitis virus. Emerg Infect Dis. (2010) 16:1093–100. doi: 10.3201/eid1607.091902, PMID: 20587180 PMC3321910

[13] Mehl C Wylezich C Geiger C Schauerte N Mätz-Rensing K Nesseler A . Reemergence of lymphocytic choriomeningitis *Mammarenavirus*, Germany. Emerg Infect Dis. (2023) 29:631–4. doi: 10.3201/eid2903.221822, PMID: 36823667 PMC9973704

[14] Rovid A. Lymphocytic choriomeningitis (2020). Available online at: https://www.cfsph.iastate.edu/Factsheets/pdfs/lymphocytic_choriomeningitis.pdf (accessed December 18, 2024)

[15] Traub E. An epidemic in a mouse colony due to the virus of acute lymphocytic choriomeningitis. J Exp Med. (1936) 63:533–46. doi: 10.1084/jem.63.4.533, PMID: 19870488 PMC2133355

[16] Mosset P Lazor-Blanchet C. Lymphocytic choriomeningitis virus infection following occupational ocular exposure. BMJ Case Rep. (2024) 17:e260966. doi: 10.1136/bcr-2024-260966, PMID: 39461842 PMC11557448

[17] Mobo BHP Rabinowitz PM Conti LA Taiwo OA. Occupational health of animal workers. Human-Animal Medicine (2010) 343–371. doi: 10.1016/B978-1-4160-6837-2.00012-9

[18] Bonthius DJ Perlman S. Congenital viral infections of the brain: lessons learned from lymphocytic choriomeningitis virus in the neonatal rat. PLoS Pathog. (2007) 3:e149. doi: 10.1371/journal.ppat.0030149, PMID: 18052527 PMC2092377

[19] Fischer SA Graham MB Kuehnert MJ Kotton CN Srinivasan A Marty FM . Transmission of lymphocytic choriomeningitis virus by organ transplantation. N Engl J Med. (2006) 354:2235–49. doi: 10.1056/NEJMoa053240, PMID: 16723615

[20] Palacios G Druce J Du L Tran T Birch C Briese T . A new arenavirus in a cluster of fatal transplant-associated diseases. N Engl J Med. (2008) 358:991–8. doi: 10.1056/NEJMoa073785, PMID: 18256387

[21] Amman BR Pavlin BI Albariño CG Comer JA Erickson BR Oliver JB . Pet rodents and fatal lymphocytic choriomeningitis in transplant patients. Emerg Infect Dis. (2007) 13:719–25. doi: 10.3201/eid1305.061269, PMID: 17553250 PMC2738461

[22] Mac Neil A Ströher U Farnon E Campbell S Cannon D Paddock CD . Solid organ transplant–associated lymphocytic choriomeningitis, United States, 2011. Emerg Infect Dis. (2012) 18:1256–62. doi: 10.3201/eid1808.120212, PMID: 22839997 PMC3414043

[23] Bonthius DJ. Lymphocytic choriomeningitis virus: an underrecognized cause of neurologic disease in the fetus, child, and adult. Semin Pediatr Neurol. (2012) 19:89–95. doi: 10.1016/j.spen.2012.02.002, PMID: 22889536 PMC4256959

[24] Pérez-Ruiz M Navarro-Marí J-M Sánchez-Seco M-P Gegúndez M-I Palacios G Savji N . Lymphocytic choriomeningitis virus–associated meningitis, Southern Spain. Emerg Infect Dis. (2012) 18:855–8. doi: 10.3201/eid1805.111646, PMID: 22515986 PMC3358079

[25] Lewis JM Utz JP. Orchitis, Parotitis and meningoencephalitis due to lymphocytic-choriomeningitis virus. N Engl J Med. (1961) 265:776–80. doi: 10.1056/NEJM196110192651604, PMID: 14464883

[26] ECDC. Factsheet for health professionals about arenavirus (2008). Available online at: https://www.ecdc.europa.eu/en/arenavirus-infection/facts (Accessed October 04, 2008).

[27] Delaine M Weingertner A-S Nougairede A Lepiller Q Fafi-Kremer S Favre R . Microcephaly caused by lymphocytic choriomeningitis virus. Emerg Infect Dis. (2017) 23:1548–50. doi: 10.3201/eid2309.170775, PMID: 28820372 PMC5572864

[28] Koroknai A Nagy A Nagy O Csonka N Mezei E Szomor K . Lymphocytic choriomeningitis virus infections in Hungary between 2017–2023—investigation of the first congenital infections. Diagnostics (Basel). (2024) 14:1436. doi: 10.3390/diagnostics14131436, PMID: 39001326 PMC11241323

[29] Wright R Johnson D Neumann M Ksiazek TG Rollin P Keech RV . Congenital lymphocytic choriomeningitis virus syndrome: a disease that mimics congenital toxoplasmosis or cytomegalovirus infection. Pediatrics. (1997) 100:e9. doi: 10.1542/peds.100.1.e9, PMID: 9200383

[30] Pencole L Sibiude J Weingertner AS Mandelbrot L Vauloup-Fellous C Picone O. Congenital lymphocytic choriomeningitis virus: a review. Prenat Diagn. (2022) 42:1059–69. doi: 10.1002/pd.6192, PMID: 35695127

[31] Barton LL Mets MB. Congenital lymphocytic choriomeningitis virus infection: decade of rediscovery. Clin Infect Dis. (2001) 33:370–4. doi: 10.1086/321897, PMID: 11438904

[32] Ferenc T Vujica M Mrzljak A Vilibic-Cavlek T. Lymphocytic choriomeningitis virus: an under-recognized congenital teratogen. World J Clin Cases. (2022) 10:8922–31. doi: 10.12998/wjcc.v10.i25.8922, PMID: 36157656 PMC9477052

[33] Negredo Antón AI de Ory Manchón F Sánchez-Seco Fariñas MP Franco Narváez L Gegúndez Cámara MI Navarro Mari JM . Diagnóstico microbiológico de arbovirosis y robovirosis emergentes. Enferm Infecc Microbiol Clin. (2015) 33:197–205. doi: 10.1016/j.eimc.2013.07.011, PMID: 24139129

[34] Emonet S Retornaz K Gonzalez J-P de Lamballerie X Charrel RN. Mouse-to-human transmission of variant lymphocytic choriomeningitis virus. Emerg Infect Dis. (2007) 13:472–5. doi: 10.3201/eid1303.061141, PMID: 17552104 PMC2725903

[35] Vilibic-Cavlek T Savic V Ferenc T Mrzljak A Barbic L Bogdanic M . Lymphocytic choriomeningitis—emerging trends of a neglected virus: a narrative review. Trop Med Infect Dis. (2021) 6:88. doi: 10.3390/tropicalmed6020088, PMID: 34070581 PMC8163193

[36] Lim J Shin SG Lee S Hwang S. Design and use of group-specific primers and probes for real-time quantitative PCR. Front Environ Sci Eng China. (2011) 5:28–39. doi: 10.1007/s11783-011-0302-x

[37] Fedele CG Negredo A Molero F Sánchez-Seco MP Tenorio A. Use of internally controlled real-time genome amplification for detection of variola virus and other orthopoxviruses infecting humans. J Clin Microbiol. (2006) 44:4464–70. doi: 10.1128/JCM.00276-06, PMID: 17065259 PMC1698395

[38] Vázquez A Herrero L Negredo A Hernández L Sánchez-Seco MP Tenorio A. Real time PCR assay for detection of all known lineages of West Nile virus. J Virol Methods. (2016) 236:266–70. doi: 10.1016/j.jviromet.2016.07.026, PMID: 27481597

[39] Davó L Herrero L Sánchez-Seco MP Labiod N Roiz D Gómez-Díaz E . Real-time RT-PCR assay to detect Granada virus and the related Massilia and Arrabida phleboviruses. Parasit Vectors. (2020) 13:270. doi: 10.1186/s13071-020-04110-5, PMID: 32471505 PMC7257231

[40] Reed LJ Muench H. A simple method of estimating fifty per cent endpoints. Am J Hyg. (1938) 27:493–7.

[41] Stephensen CB Blount SR Lanford RE Holmes KV Montali RJ Fleenor ME . Prevalence of serum antibodies against lymphocytic choriomeningitis virus in selected populations from two U.S. cities. J Med Virol. (1992) 38:27–31. doi: 10.1002/jmv.1890380107, PMID: 1402829

[42] Becker SD Bennett M Stewart JP Hurst JL. Serological survey of virus infection among wild house mice (*Mus domesticus*) in the UK. Lab Anim. (2007) 41:229–38. doi: 10.1258/002367707780378203, PMID: 17430622

[43] Knust B. Exposure to lymphocytic choriomeningitis virus, New York, USA. Emerg Infect Dis. (2011) 17:1324–5. doi: 10.3201/eid1707.101349, PMID: 21762607 PMC3381384

[44] Williams SH Che X Garcia JA Klena JD Lee B Muller D . Viral diversity of house mice in New York City. MBio. (2018) 9:e01354-17. doi: 10.1128/mBio.01354-17, PMID: 29666290 PMC5904411

[45] Lledó L Serrano JL Giménez-Pardo C Gegúndez I. Wild red foxes (*Vulpes vulpes*) as sentinels of rodent-borne hantavirus and lymphocytic choriomeningitis virus in the province of Soria, Northern Spain. J Wildl Dis. (2020) 56:658–61. doi: 10.7589/2019-09-239, PMID: 32011204

[46] Martos Fernández E García Gestoso ML Marín Pérez J Jiménez Alés R Catalán Muñoz M Romero Cachaza J . Encephalitis due to the lymphocytic choriomeningitis virus. An Esp Pediatr. (1996) 44:512–4. 8928978

[47] De Ory F Gegúndez MI Fedele CG Sánchez-Seco MP. Virus Toscana, West Nile y de la coriomeningitis linfocitaria como causantes de meningitis aséptica en España. Med Clin (Barc). (2009) 132:587–90. doi: 10.1016/j.medcli.2008.10.057, PMID: 19375119

[48] Childs JE Glass GE Korch GW Ksiazek TG Leduc JW. Lymphocytic choriomeningitis virus infection and house mouse (*Mus Musculus*) distribution in urban Baltimore. Am J Trop Med Hyg. (1992) 47:27–34. doi: 10.4269/ajtmh.1992.47.27, PMID: 1636880

[49] Tagliapietra V Rosà R Rossi C Rosso F Hauffe HC Tommasini M . Emerging rodent-borne viral zoonoses in Trento, Italy. EcoHealth. (2018) 15:695–704. doi: 10.1007/s10393-018-1335-4, PMID: 29796719

[50] Lledó L Gegúndez MI Saz JV Bahamontes N Beltrán M. Lymphocytic choriomeningitis virus infection in a province of Spain: analysis of sera from the general population and wild rodents. J Med Virol. (2003) 70:273–5. doi: 10.1002/jmv.10389, PMID: 12696116

[51] Lledó L Giménez-Pardo C Gegúndez MI. Screening of forestry workers in Guadalajara Province (Spain) for antibodies to lymphocytic choriomeningitis virus, hantavirus, *Rickettsia* spp. and *Borrelia burgdorferi*. Int J Environ Res Public Health. (2019) 16:4500. doi: 10.3390/ijerph16224500, PMID: 31731580 PMC6888197

[52] Sarli J Lutermann H Alagaili AN Mohammed OB Bennett NC. Seasonal reproduction in the Arabian spiny mouse, *Acomys dimidiatus* (Rodentia: Muridae) from Saudi Arabia: the role of rainfall and temperature. J Arid Environ. (2016) 124:352–9. doi: 10.1016/j.jaridenv.2015.09.008

[53] Dantas MRT Souza-Junior JBF Castelo T d S Lago AE d A Silva AR. Understanding how environmental factors influence reproductive aspects of wild myomorphic and hystricomorphic rodents. Anim Reprod. (2021) 18:e20200213. doi: 10.1590/1984-3143-ar2020-0213 33936293 PMC8078862

[54] Sipari S Khalil H Magnusson M Evander M Hörnfeldt B Ecke F. Climate change accelerates winter transmission of a zoonotic pathogen. Ambio. (2022) 51:508–17. doi: 10.1007/s13280-021-01594-y, PMID: 34228253 PMC8800963

[55] Evander M Ahlm C. Milder winters in Northern Scandinavia may contribute to larger outbreaks of haemorrhagic fever virus. Glob Health Action. (2009) 2:2020. doi: 10.3402/gha.v2i0.2020, PMID: 20052429 PMC2799289

[56] Hinman AR Fraser DW Douglas RG Bowen GS Kraus AL Winkler WG . Outbreak of lymphocytic choriomeningitis virus infections in medical center personnel1. Am J Epidemiol. (1975) 101:103–10. doi: 10.1093/oxfordjournals.aje.a112076 1092154

[57] Knust B Ströher U Edison L Albariño CG Lovejoy J Armeanu E . Lymphocytic choriomeningitis virus in employees and mice at multipremises feeder-rodent operation, United States, 2012. Emerg Infect Dis. (2014) 20:240–7. doi: 10.3201/eid2002.130860, PMID: 24447605 PMC3901486

[58] Shek W. Lymphocytic choriomeningitis virus In: Waggie K Kagiyama N Allen A Nomura T, editors. Manual of microbiologic monitoring of laboratory animals. Bethesda (MD): US Department of Health and Human Services (1994). 35–42.

[59] Buchheister S Bleich A. Health monitoring of laboratory rodent colonies—talking about (R)evolution. Animals. (2021) 11:1410. doi: 10.3390/ani11051410, PMID: 34069175 PMC8155880

[60] CDC. Update: Interim guidance for minimizing risk for human lymphocytic choriomeningitis virus infection associated with pet rodents (2005). Available online at: https://www.cdc.gov/mmwr/preview/mmwrhtml/mm5432a3.htm (accessed May 26, 2025) 16107785

[61] Cordey S Sahli R Moraz ML Estrade C Morandi L Cherpillod P . Analytical validation of a lymphocytic choriomeningitis virus real-time RT-PCR assay. J Virol Methods. (2011) 177:118–22. doi: 10.1016/j.jviromet.2011.06.018, PMID: 21763351

[62] McCausland MM Crotty S. Quantitative PCR technique for detecting lymphocytic choriomeningitis virus in vivo. J Virol Methods. (2008) 147:167–76. doi: 10.1016/j.jviromet.2007.08.025, PMID: 17920702 PMC2330273

[63] Linkowska K Bogiel T Lamperska K Marszałek A Starzyński J Szylberg Ł . Commercially available SARS-CoV-2 RT-qPCR diagnostic tests need obligatory internal validation. Sci Rep. (2023) 13:6991. doi: 10.1038/s41598-023-34220-w, PMID: 37117538 PMC10144901

[64] Chung Y-S Lee N-J Woo S-H Kim J-M Kim H-M Jo HJ . Validation of real-time RT-PCR for detection of SARS-CoV-2 in the early stages of the COVID-19 outbreak in the Republic of Korea. Sci Rep. (2021) 11:14817. doi: 10.1038/s41598-021-94196-3, PMID: 34285290 PMC8292370

