# Supplementary material for: Development of a new quantitative RT-PCR to detect lymphocytic choriomeningitis virus
Source: Front Vet Sci. 2025 Dec 24;12:1651039. doi: 10.3389/fvets.2025.1651039 (PMC12777075; doi:10.3389/fvets.2025.1651039)
Supplement: Supplementary file 1 [file Table_1.DOCX]

| **Virus** | **Family** |
| --- | --- |
| Junín Virus or Argentine hemorrhagic fever | *Arenaviridae* |
| Guanarito Virus or Venezuelan hemorrhagic fever | *Arenaviridae* |
| Sabiá Virus or Brazilian hemorrhagic fever | *Arenaviridae* |
| Tacaribe Virus | *Arenaviridae* |
| Machupo Virus or Bolivian hemorrhagic fever | *Arenaviridae* |
| Lassa Virus (Nigeria strain) | *Arenaviridae* |
| Lassa Virus (Josiah strain) | *Arenaviridae* |
| Zika Virus | *Flaviviridae* |
| Japanese Encephalitis Virus | *Flaviviridae* |
| San Louis Encephalitis Virus | *Flaviviridae* |
| West Nile Virus Lineaje I | *Flaviviridae* |
| West Nile Virus Lineaje II | *Flaviviridae* |
| Usutu Virus | *Flaviviridae* |
| Tick-borne Encephalitis Virus | *Flaviviridae* |
| Cytomegalovirus | *Herpesviridae* |
| Herpes Simplex Virus 1 | *Herpesviridae* |
| Herpes Simplex Virus 2 | *Herpesviridae* |
| Rabies Virus | *Rhabdoviridae* |
| Human Parvovirus B19 | *Parvoviridae* |
| Western Equine Encephalitis | *Togaviridae* |
| Eastern Equine Encephalitis | *Togaviridae* |
| Venezuelan Equine Encephalitis | *Togaviridae* |
| Rubella | *Togaviridae* |
| Toscana Virus | *Togaviridae* |
| Enterovirus 71 | *Picornaviridae* |
| Echovirus 9 | *Picornaviridae* |
| Echovirus 30 | *Picornaviridae* |
| Enterovirus D68 | *Picornaviridae* |

**Table S1**: List of other arenaviruses and other encephalitis-producing viruses used in this study to test the specificity of the developed LCMV RT-qPCR.
