# Supplementary material for: Development of a new quantitative RT-PCR to detect lymphocytic choriomeningitis virus
Source: Front Vet Sci. 2025 Dec 24;12:1651039. doi: 10.3389/fvets.2025.1651039 (PMC12777075; doi:10.3389/fvets.2025.1651039)
Supplement: Supplementary file 2 [file Table_2.DOCX]

| **Name** | **Sequence (5’-3’)** |
| --- | --- |
| LCM 1+ | TCWCKATGTAKGGCCAYCCTTCMCC |
| LCM 1- | GTYAARAGCTTYCARTGGACRCAR |
| LCM 2+ | TTKATRCTRGAYTGCTGYTCAGT |
| LCM 2- | AACAAYCAVTTYGGMACHATGCC |

**Table S2:** Primers used in the conventional nested RT-PCR for LCMV detection
